# Supplementary material for: Are carbon emissions trading and green financial instruments synergistic? -Comprehensive quantitative research based on content analysis
Source: PLoS One. 2024 Mar 7;19(3):e0298601. doi: 10.1371/journal.pone.0298601 (PMC10919601; doi:10.1371/journal.pone.0298601)
Supplement: S1 Data — (ZIP) [file pone.0298601.s001.zip › Data/地级市Z_F工作报告.zip!/╝¬┴╓/│ñ┤║╩╨/└┤╫╘╣1⁄2╓┌║┼í╛┬φ┐╦╩2╛▌═°í┐_2019.docx]

各位代表：

　　现在，我代表市政府，向大会报告工作，请予审议，并请市政协委员提出意见。

　　一、2018年工作回顾

　　2018年是全面贯彻党的十九大精神的开局之年，也是长春振兴发展进程中极不平凡的一年。一年来，在省委、省政府和市委正确领导下，在市人大、市政协监督支持下，我们深入学习贯彻习近平新时代中国特色社会主义思想和党的十九大精神，统筹推进“五位一体”总体布局，协调推进“四个全面”战略布局，全面践行新发展理念，按照“打先锋、站排头”要求，解放思想、抢抓机遇，迎难而上、扎实工作，较好完成了全年各项目标任务。

　　（一）落实高质量发展要求，经济整体素质稳步提高。全市经济继续保持平稳较快增长态势。预计地区生产总值增长7%以上，总量迈上7000亿元台阶，占全省经济比重达到45%，比上年提高2.3个百分点。地方级财政收入完成478亿元，增长6.2%。规模以上工业增加值增长8%以上。实施亿元以上项目1206个，固定资产投资增长7%。社会消费品零售总额完成3028亿元，增长7%。城乡居民收入与经济增长基本同步。

　　积极推动汽车、轨道客车、农产品加工三大支柱产业优化升级，奥迪Q工厂、中粮聚乳酸等11个投资超10亿元工业项目竣工投产。加快培育先进装备制造、光电信息、生物及医药健康、新能源汽车、新材料、大数据六大战略性新兴产业，战略性新兴产业产值增长15.9%。启动数字长春建设，积极培育共享经济、数字经济、平台经济，促进互联网、大数据、人工智能与制造业深度融合，华为云计算、浪潮大数据等重点项目顺利推进。

　　成功举办汽博会、农博会、电影节等重要展会，会展业交易额增长11%。全年接待游客8900万人次，增长15%。龙嘉机场旅客吞吐量增长11.2%。欧亚汇集等4个商业综合体建成开业。5家域外金融机构落户长春。

　　出台实施乡村振兴战略规划。新建高标准农田73.2万亩，推广保护性耕作381万亩。高效特色农业、园艺设施农业、精品畜牧业稳步发展。

　　（二）深化改革开放，振兴发展动能持续释放。深化“放管服”改革，企业开办时间压减到3个工作日，工业项目审批时间压减到15个工作日，97%的行政审批和公共服务事项实现“最多跑一次”。启动“标准地+承诺制”改革试点，“多证合一”“证照分离”改革全面推开，“双随机、一公开”监管方式实现全覆盖。

　　国资国企、产权制度、投融资、农业农村、教育卫生等领域改革持续推进，探索形成了产业扶贫创新、盘活集体土地等长春特色的改革经验。完善落实民营经济支持措施，全年新登记民营企业3.6万户，总量达到19.7万户，民营经济占GDP比重达到51%。

　　深入实施创新驱动发展战略，新增国家高新技术企业315户、科技型“小巨人”企业283户。完成技术合同交易额323.5亿元，增长52%。以长春新区为核心的长吉图开发开放先导区获批国家级科技成果转移转化示范区。落实“人才新政”20条，7.8万名高校毕业生留长创业就业，增长23.8%。

　　主动融入“一带一路”建设，纽伦堡枢纽及海外仓正式启用，长春获批设立汽车整车进口口岸和跨境电子商务综合试验区。加快拓展多元化市场，进出口总额增长10.4%。新引进央企和国内500强企业14户，实际利用内资、直接利用外资分别增长16%和9%。

　　全面落实全省“一主、六双”产业空间布局，稳步实施长吉一体化、长春公主岭同城化。持续推进哈长城市群建设，积极推动与天津、杭州对口合作。成功开展“外国使节团走进长春”等对外交往活动，城市影响力、知名度稳步提升。

　　（三）完善功能品质，城市现代化建设水平全面提升。强化规划引领作用，城市设计、城市总体规划、“多规合一”空间规划三项国家试点取得阶段性成果。

　　历时三年的旧城改造提升工程顺利完成，三环以内166平方公里城区旧貌换新颜。伊通河中段“五岛十园”景观、22座驿站、33公里绿道建成开放。水文化生态园、道台衙门博物馆、奥林匹克公园投入使用，侵华日军“第一〇〇部队”遗址园完成抢救加固并对外开放。新植街路58条，新建大宗绿地58块，植树造林4972公顷。

　　地铁2号线、轻轨北湖线载客运营，轨道交通三期规划正式获批。龙嘉机场T2航站楼、综合交通枢纽一期北区及火车站南广场综合交通换乘中心投入使用。提升改造维修路桥68条（座），“两横三纵”快速路部分续建工程竣工通车，长春经济圈环线高速公路启动建设。深入开展“走遍长春”专项行动，城市精细化管理水平稳步提升。

　　（四）加快绿色发展，生态环境质量持续改善。坚决贯彻习近平生态文明思想，全面推动中央和省环保督察反馈问题整改，严守生态、耕地保护、城市开发边界三条红线，努力构建生态环保长效机制。

　　深入落实河（湖）长制，提标改造9座污水处理厂，禁养区内规模养殖场全部关闭搬迁，伊通河、饮马河水质改善向好。建成区75处黑臭水体基本消除黑臭，长春获评“2018年城市黑臭水体治理示范城市”。石头口门、新立城两大水源地一级保护区实现全封闭管理。“全国水生态文明试点城市”通过国家验收。

　　淘汰20吨以下燃煤锅炉2435台，430所农村学校完成火炉取暖替代改造，坚决禁止禁烧区秸秆露天焚烧。从严整治工业烟粉尘、建筑工地扬尘、汽车尾气，空气质量优良天数比上年增加46天。扎实推进林地清收、矿山修复和退耕还草还湿。城市垃圾分类试点范围稳步扩大。

　　（五）办好民生实事，群众幸福指数日益提高。努力增加民生投入，幸福长春行动计划确定的98件实事全部兑现。

　　扎实精准推进脱贫攻坚战。启动实施产业扶贫项目155个，新建改造农村安全饮水工程101处，改造贫困户危房2934户，贫困村光纤、4G网络基本实现全覆盖。15个贫困村出列、1535户贫困户脱贫。

　　实施更加积极的就业政策，城镇新增就业11.4万人。继续实施城乡居民增收“暖流计划”，受益群众人均增收524元。城镇职工、城乡居民基本养老金分别提高6%和18%，失业保险金提高20.5%，工伤待遇标准提高5%。

　　上调低保对象基本医疗住院救助比例。为5.1万名残疾人提供精准康复服务，“三无一靠”成年重度残疾人补贴标准提高1.5倍。为9752名特殊和困难老人购买居家养老服务。城区居民基本殡葬费用全部免除。

　　改造棚户区住宅1.02万套，安置超期回迁居民2202户，完成“无籍房”确权登记12.9万户、1137万平方米。115个老旧小区物业建立长效管理机制。

　　深入创建“公交都市”，新增更新公交车辆400台。新建农村公路420公里。农村人居环境整治扎实推进，20个村获评省级“美丽乡村”。

　　新增义务教育学校6所、普惠性幼儿园18所。小学生课后免费托管服务“蓓蕾计划”被中央改革办作为改革经验向全国推广。成功举办第二届长春国际马拉松赛。

　　（六）创新社会治理，社会保持和谐稳定。自觉接受市人大及其常委会法律监督、市政协民主监督，主动接受社会和舆论监督，认真听取各民主党派、工商联以及无党派人士意见建议。

　　加强群众信访工作。提高局长接待日、市长公开电话工作水平。积极开展“电视问政”，聚焦旧城改造提升、农村水利、河（湖）长制、文明城创建，又解决了138件关乎民生发展的突出问题。深入开展“肃行风、转作风、提能力”行动，全面强化政府监管和行业自律，着力解决医疗卫生、出租车管理等领域损害群众利益的突出问题。拆除违建“大棚房”670处、9.2万平方米。严控政府隐性债务，全力防范金融风险。

　　建设“平安长春”。扎实开展扫黑除恶专项斗争，命案破案率100%，重大有影响案件全部告破，在全国358个地级以上城市群众安全感排名前移至第16位。公共安全视频监控联网应用“雪亮工程”如期竣工。深入推进安全生产专项整治，安全生产事故起数、遇难人数连续66个月“双下降”。

　　积极培育践行社会主义核心价值观，扎实推进全国文明城市创建。推动军民融合深度发展。驻长部队全面停止有偿服务任务基本完成。民族团结、宗教和睦良好局面进一步巩固。

　　过去一年，“两学一做”学习教育常态化制度化扎实推进。对照习近平总书记对德惠“6·3”特大火灾事故、侵华日军“第一〇〇部队”遗址、长生问题疫苗案件重要指示批示开展“回头看”，确保总书记重要指示批示全面落实。坚持从严治政，全面彻底肃清孙政才、苏荣、王珉流毒影响。认真落实中央八项规定精神。深入开展干部作风大整顿。开展解放思想推动长春高质量发展大讨论。严格依法行政。加强审计监督。驰而不息推进党风廉政建设。

　　工业稳增长和转型升级、老工业基地调整改造、深化商事制度改革落实事中事后监管、公立医院综合改革、“审批不见面、办事不求人”政务服务新模式以及九台区秸秆综合利用新样板等方面工作受到国务院通报表扬。国家安全、统计、供销、气象、地震、档案、人防、公积金、地方志、红十字等领域工作也取得了新进展。各位代表，过去一年长春经济社会发展取得的成绩，是深入贯彻落实习近平新时代中国特色社会主义思想和党的十九大精神的结果，是省委、省政府和市委正确领导的结果，是市人大、市政协监督支持的结果，是全市广大干部群众团结奋斗的结果。在此，我代表市政府，向全市人民、人大代表、政协委员，向各民主党派、工商联、人民团体，向驻长中省直单位、人民解放军和武警官兵，向所有关心、支持长春发展的各界人士和国际友人，致以崇高的敬意和衷心的感谢！

　　肯定成绩的同时也要清醒看到，我们的工作还存在体制机制、经济结构、开放合作、思想观念四大短板，突出表现在思想解放力度还不够大，市场意识、法治意识、创新意识、担当意识、责任意识仍需增强；改革还不够深入，制约长春全面振兴、全方位振兴的体制机制矛盾依然存在；市场化程度还不高，民营经济发展遇到一些困难；战略性新兴产业和现代服务业规模较小，新产业、新业态培育不够快；对外开放水平不高，区域发展不够协调，县域经济增长趋缓；民生改善与人民群众的期盼还有差距，社会文明程度需要进一步提高；政府职能转变还不到位，廉政建设和作风建设仍需加强。对此，我们将直面矛盾、正视问题，采取有效措施，切实加以解决，不负人民重托！

　　二、2019年重点工作

　　今年是新中国成立70周年，是贯彻落实习近平总书记在深入推进东北振兴座谈会上的重要讲话和视察东北时的重要指示精神，推动长春全面振兴、全方位振兴的关键一年。我们必须始终坚持以“立足吉林、放眼全国、走向世界”的战略眼光，奋力谱写振兴发展的新篇章。2019年政府工作的总体思路是:高举习近平新时代中国特色社会主义思想伟大旗帜，全面贯彻党的十九大、习近平总书记在深入推进东北振兴座谈会上的重要讲话和视察东北时的重要指示精神，按照中央、全省经济工作会议和市委十三届六次全会部署，统筹推进“五位一体”总体布局，协调推进“四个全面”战略布局，坚持稳中求进工作总基调，坚持新发展理念，坚持推动高质量发展，坚持以供给侧结构性改革为主线，坚持深化市场化改革、扩大高水平开放，全力打好“三大攻坚战”，坚决落实“六稳”要求，统筹推进稳增长、促改革、调结构、惠民生、防风险工作，不断强化“打先锋、站排头”意识，认真落实省委“三个五”发展战略，加快建设东北亚区域性中心城市，为实现长春全面振兴、全方位振兴奠定坚实基础，以优异成绩迎接中华人民共和国成立70周年。

　　今年全市经济发展主要预期目标是：地区生产总值增速保持在7%以上，规模以上工业增加值增长8%以上，固定资产投资增长8%以上，地方级财政收入突破500亿元、增长5%以上，城乡居民收入与经济增长基本同步。

　　重点做好八个方面工作：

　　（一）坚持稳中求进，推动经济平稳健康增长。争取和整合政策激活力，招引和建设项目增实力，壮大和提升产业添能力，培育和扶持企业挖潜力，夯实基础、提质增效、行稳致远。

　　牢牢把握国家战略机遇。积极参与国家和省各种政策制定，争取重大战略、重大政策、重大项目、重大平台、重大工程。积极申建自贸区、中韩国际合作示范区，争创国家自主创新示范区。

　　牢牢抓住重大项目。全面提高项目策划、推介、洽谈、签约、开工、投产的国际化、现代化、组织化、专业化、平台化水平。深入实施“项目建设年”活动，确保全年落实亿元以上项目1200个以上。集中精力抓好红旗升级改造、一汽丰越扩能改造和金赛药业、百克疫苗等百个投资超10亿元产业项目，力争早建成、早投产、早见效。

　　牢牢强化招商引资。围绕产业链、产业群、产业生态的功能取向，供应链、制造链、服务链的价值取向，城乡品位、品质、品牌、时尚的需求取向，人文底蕴、资源禀赋、产业基础、科教医体旅养的潜力取向，进一步完善招商引资的项目生成机制和政策体系、组织体系、服务体系，重点引进世界500强企业、央企、民营上市公司、独角兽企业、高新技术企业，推动招商引资规模、结构、质量、效益稳步提升。全年实际利用内资、直接利用外资均增长10%以上，产业项目占比明显提高。

　　激发各类市场主体活力。建立全方位的企业服务体系，实施“百强民营企业”、百户“专精特新”中小企业培育工程。深化民营经济发展改革示范城市建设，尽快出台促进民营经济高质量发展的相关政策。全年新登记民营企业2万户以上，民营经济主营业务收入增长8.5%以上。

　　（二）突出质量效益，做大做强做优实体经济。坚定不移把振兴发展的着力点放在壮大实体经济上，积极改造提升传统产业，大力培植新兴产业，加快建立现代经济体系。

　　加快产业高质量发展。着力打造汽车、轨道客车、农产品加工三大支柱产业集群。认真落实千亿级新兴产业培育计划，六大战略性新兴产业产值增长15%以上。深化“十三五”国家服务业综合改革试点，推进“互联网+医疗、养老、教育、文化、体育、旅游”等生活性服务业，大力发展产业链金融、现代物流、工业设计、研发孵化、工业地产等生产性服务业。充分挖掘长春人文积淀、影视底蕴、高校资源，大力发展网络文学、影视制播、新闻出版、直播平台、动漫游戏、电子竞技等文化创意产业，文创产业增加值增长10%以上。高品质打造南部新城、长春西站、北人民大街等现代服务业集群。开展“千企促销专项行动”和“放心消费在长春”创建活动，积极释放内需潜力。

　　突出龙头企业带动引领。全力支持一汽集团及其合资企业发展。以一汽为全方位开发开放合作平台，积极推动一汽扩大与世界500强企业、央企、民企、高校、科研院所合作，促进汽车产业与战略性新兴产业深度融合。大力发展汽车后市场服务，围绕售车、修车、改车、饰车、赛车、二手车、再制造构建产业体系，通过客户群、专业俱乐部、国际汽车赛事，全面提升“汽车城”影响力。以欧亚、长客、皓月、亚泰、大陆汽车电子等各行业龙头企业为价值平台，深度整合产业链金融、信息、科技、人才等要素服务体系，向上下游延伸产业链，扩大相关生产性服务业比重。

　　增强科技创新驱动力。扎实推进国家创新型城市和吉林中部创新转型核心区建设，滚动实施重大科技攻关和重大科技成果转化“双十工程”，国家高新技术企业、科技型“小巨人”企业各新增200户以上。依托在长企业、高校和科研院所，加强与国内外知名高校、科研机构、孵化器、投资机构的深度融合，提高“政产学研用金介”协同创新平台的实用性、实效性，努力营造国内一流的科技创新生态环境。完善科技大市场、科技金融创新中心功能，加快建设创新创业服务支撑体系，技术合同交易额力争增长20%以上。建设国家级“双创”示范基地，新建省级以上双创基地5个，在孵企业总数达到1.65万户。长春新区国家知识产权运营公共服务平台北方平台投入运营。深入实施质量强市战略，全面开展质量提升行动。加快完善人才激励机制，在创新创业、安家落户、子女教育、医疗养老等方面，加大对长春发展有突出贡献人才的服务力度。持续推进“高校毕业生留长创业就业”工程，当年落户人数力争突破8万人，增长5%以上。弘扬工匠精神，培养高素质劳动者和技术技能人才。

　　（三）深化改革开放，激发经济社会发展活力。全面贯彻习近平总书记在庆祝改革开放40周年大会上的重要讲话精神，以改革破除一切制约振兴发展的思想羁绊和体制障碍，以创新引领社会活力竞相迸发。

　　打造一流营商环境。对照国家营商环境评价体系的23类124项指标，顶层设计、全面统筹、系统提升，力争把营商环境质量提升到全国一流水平。进一步优化政务环境，深化“审批不见面、办事不求人”的改革实践，新开办企业工商注册审批用时减到30分钟以内，一般性工业项目审批用时减到5个工作日以内。进一步优化市场环境，充分发挥市场决定性作用，在资源配置、公共服务中注重公平、公正、公开，强化信用监管促进企业诚信守法经营。进一步优化法治环境，启动实施“互联网+监管”改革，推动市场监管等重点领域综合执法，综合运用法律、经济、行政、文化等各种有效手段提高监管效能，实现德治与法治的有机统一。进一步优化经营环境，全面落实国家减税降费措施，尽快实现涉企收费统一收缴、清单之外无收费，力推水电气热通讯等公共服务降价降费，管线接入时间压缩一半以上。进一步优化政策环境，围绕重点产业、招商引资、创新驱动、公共服务、城市管理、生态保护等重点领域深化政策创新，打造东北最优的政策体系。进一步优化社会环境，构建“亲”“清”新型政商关系，在全社会营造尊商、亲商、富商、安商的浓厚氛围，让企业家和创业者经济上得实惠、社会上有地位。

　　推进重点领域改革。按照“巩固、增强、提升、畅通”八字方针扎实推进供给侧结构性改革，巩固“三去一降一补”成果，增强微观主体活力，提升产业链水平，畅通经济循环。深入推进国资国企改革，以管资本为主强化国资监管，加快建立现代国有企业制度，确保国有投资精准有效、国有资产保值增值。下力气解决厂办大集体改革等历史遗留问题。完成市级政府机构改革，形成职责明确、依法行政的政府治理体系，增强政府公信力和执行力。统筹推进文化、教育、医疗、养老服务、安全生产、食品安全等领域改革。

　　提高对外开放水平。加快空港经济开发区建设，启动建设跨境电子商务综合试验区，增强兴隆综合保税区聚集外资能力，提升国际货运包机、货运班列运营质量，增开国际客运航线。稳步提高机电、高新技术、农产品等优势产品出口份额，全年进出口总额增长6%以上。深化与德日韩全方位合作，在产业以及文化、教育、医疗、体育、养老服务、城市管理等方面加大交流合作力度。积极开展与欧美、俄罗斯、朝鲜、蒙古合作交流，加强同“一带一路”沿线国家地区联系沟通，巩固提高与国际友城、友好合作城市交流合作的实效性。

　　（四）坚持问题导向，打好“三大攻坚战”。统筹推进、持续用力、真抓实干，打好打赢“三大攻坚战”。

　　打好防范化解重大风险攻坚战。加强政府性债务管控，严控增量。整顿不规范融资担保，严控政府隐性债务。按照国务院要求，一律按不低于5%的幅度压减一般性财政支出。加强金融风险源头管控，严厉打击违法违规金融活动，妥善处置应对P2P网络借贷等风险。

　　打好精准脱贫攻坚战。严格执行“两不愁三保障”标准，进一步强化教育、就业、健康、保障脱贫。启动实施产业扶贫项目142个，新建改造农村安全饮水工程29处，确保7个贫困村出列、1538户贫困户脱贫。加强脱贫攻坚与乡村振兴统筹衔接，切实防止返贫和产生新的贫困，确保脱贫成果经得起历史和实践检验。

　　打好污染防治攻坚战。牢固树立“绿水青山就是金山银山”理念，坚决打赢蓝天、碧水、黑土地、青山、湿地“五大保卫战”，让长春的天更蓝、山更绿、水更清。坚决禁止禁烧区秸秆露天焚烧。从严整治柴油货车污染。597台20吨以下燃煤锅炉全部淘汰。深入落实河（湖）长制，饮马河、伊通河基本消除劣五类水体。石头口门、新立城两大水源地一级保护区1.9万亩耕地退耕还草还湿。80%的公共机构、260个居民小区开展垃圾分类试点。秸秆综合利用率、畜禽粪污综合利用率分别达到87%和75%。修复三北防护林，营造林4500公顷。统筹产业生态化、生态产业化，加快发展低碳经济、循环经济、绿色经济。

　　（五）注重科学统筹，推动区域协调发展。落实全省全方位对口合作、多层次战略合作、紧密型专业合作、前瞻性共建合作、开放性国际合作要求，构建长春振兴发展新优势。

　　促进市域板块竞相发展。新区、开发区要牢牢坚持项目立区、产业兴区、科技强区，提高投资强度、科技贡献率和全要素生产率，在深化改革开放、优化营商环境、聚集新兴产业、激发创新创业等方面争创一流。城区要充分依托人文底蕴、校所资源、产业基础、生态本色，大力发展特色街区、双创基地、现代服务业，不断提升产业层次、宜居水平、生态层级。县域要以乡村振兴战略为总抓手，加大招商引资力度，在产业园区、主导产业、富民项目、特色经济、生态功能区建设上求突破。

　　推动区域协同发展。发挥省会城市核心辐射带动作用，落实东中西“三大板块”战略和“一主、六双”产业空间布局。积极推动长吉一体化，共建“长吉大都市区”。推进长春公主岭同城化，加快基础设施互联互通。

　　积极开展对口合作。全面对接京津冀协同发展、长江经济带发展、粤港澳大湾区建设等国家战略，力争引进落位一批合作项目。深化与天津、杭州对口合作，加快建设津长产业合作园、吉浙产业园等共建园区，力争引进大型企业20户以上。落实哈长城市群发展规划，加快建设哈长一体化发展示范区。

　　（六）推动乡村振兴，提升“三农”工作水平。全面落实乡村振兴战略规划，按照产业兴旺、生态宜居、乡风文明、治理有效、生活富裕总要求，推动农业全面升级、农村全面进步、农民全面发展。

　　发展优质特色绿色农业。开展率先实现农业现代化攻坚行动，持续深化农业供给侧结构性改革，优化现代农业产业、生产、经营三大体系。积极调整种植结构，大力发展“菌药花菜瓜果”优质高效农业，打造特色农产品品牌，经济作物达到160万亩。深入创建“全国绿色有机农业示范市”，新建绿色有机示范园区20个，新增绿色有机面积95万亩。建设40个无抗养殖基地。新建高标准农田70万亩。稳步提高农业经营主体的集中度和整体实力。综合农机化率达到90%。发展农村电商、乡村旅游、田园综合体，促进农村各产业融合发展。

　　推动农村全面发展进步。探索培育产业特色鲜明、人文气息浓厚、生态环境优美的特色小镇5个以上。深入实施农村人居环境整治三年行动计划，因地制宜解决好生活垃圾、污水等问题。落实全省“百村引领、千村示范、万村整治”工程，打造15个高标准宜居样板村。推进农村“厕所革命”，改厕3万户。建设“四好农村路”150公里。解决3.9万农村居民饮水安全问题。开展平安乡村建设，完善农村社会矛盾纠纷化解机制，健全乡村治理体系。开展新型职业农民培育工程，全年培训6000人次。

　　（七）突出文化引领，建设宜居宜业城市。坚持以强烈的文化意识为引领，高水平规划、高质量建设、高标准管理，加快打造“让本地人自豪、外地人向往”的现代化城市。

　　突出“一张蓝图”，塑造城市特色。深入开展城市设计、城市总体规划、“多规合一”空间规划国家试点，高水平编制新一轮城市总体规划。完善伊通河城区段绿带，拆除三环以内零散棚户区30万平方米，新建续建4座公园，着力提高城区道路、单位庭院、居民小区园林绿化水平。扩大乔灌木种植面积，新增绿地210公顷。

　　坚持“双核带动”，提升城市能级。全面推进长春新区、南部新城、长春西站等重点区域开发建设。保护修缮历史建筑，改造提升红旗绿色智能小镇、长影步行街、长拖老厂区等文化特色街区。加快实施地铁2号线东西延线、轻轨3号线东延工程，全面开展第三轮轨道交通建设。完善“两横三纵”快速路，续建吉林大路快速路、长春经济圈环线高速公路，加快抚长高速人民大街出口改移。推进第六净水厂建设。新建改造地下管网240公里，新增供热面积300万平方米。

　　强化“四化治理”，提高管理水平。对标国内外发达城市管理标准体系，推动城市管理人性化、科学化、精细化、现代化。加快建立集卫星、无人机、视频监控、执法巡逻、群众举报于一体的城市、环境、自然资源监管体系，加强道路交通、建筑工地、违法建筑、市场秩序、市容环境、牌匾广告等领域专项整治，让城市更整洁、更有序、更亮丽、更文明。

　　（八）坚持以人为本，更好保障改善民生。牢固树立以人民为中心的发展思想，着力解决好人民群众反映强烈的突出问题，使人民群众有更多、更直接、更实在的获得感、幸福感、安全感。

　　努力改善民生状况。落实幸福长春行动计划，办好100件民生实事。实施“暖流计划”，促进城乡居民持续稳定增收。着力扩大就业，新增城镇就业9万人，农村剩余劳动力转移就业100万人次。加大对残疾人、城镇困难人员就业援助力度。开展农民工工资“精准支付”行动。落实全民参保计划，推动社保扩面征缴。进一步提高医疗、养老、失业、工伤保险待遇水平。开展全国居家和社区养老服务改革试点。完善社会救助体系，保障城乡生活困难人员基本生活。完成“无籍房”确权登记850万平方米以上，力争用两年时间基本消除“无籍房”。改造棚户区5300户，安置超期回迁居民3000户。新建改建城市公厕200座以上。加快“公交都市”创建，新增更新公交车辆400台，提标改造三环以内主要街路候车亭。积极推动政务服务“一网通办”“全程代办”。为民服务探索免交证明，便民服务走进“刷脸时代”。

　　大力发展社会事业。优先发展教育事业，新建4所义务教育学校，新增15所普惠性幼儿园。加强与东北师大等高校合作，推动教育内涵式发展。全面整顿规范无资质幼儿园、民办教育机构。推进健康长春建设，积极实施为民惠民医改，增加医保低自付、慢性病门诊治疗病种，推动分级诊疗、跨省异地就医结算。深入实施“文化兴市”战略，不断提升公共文化服务均等化水平。认真做好央视春晚长春分会场服务保障。广泛开展全民健身。办好第三届长春国际马拉松赛。具备条件的中小学校定时向社会开放体育设施。高标准承办举办东博会、汽博会、农博会、消夏节、冰雪节等百场节庆会展活动。积极践行社会主义核心价值观，高标准、常态化创建全国文明城市。

　　切实加强社会治理。学习推广新时代“枫桥经验”，积极推进基层社会治理现代化。创建“国家安全发展示范城市”，健全突发事件监测预警体系和应急响应机制，坚决预防重特大事故。创建“国家食品安全示范城市”，全面强化食品药品监管，确保人民群众饮食用药安全。加强社会治安综合治理，严厉打击各类违法犯罪活动，深入推进扫黑除恶专项斗争。创新信访工作方式，发挥局长接待日、市长公开电话作用，依法及时有效解决群众合理诉求。深入开展“肃行风、转作风、提能力”行动，从严整治损害群众利益的突出问题。全面推进依法治市。完善公共法律服务体系。落实普法责任制。加强民族宗教工作。全力支持军队和国防建设，推进军民融合深度发展，争创全国双拥模范城“九连冠”。

　　三、加强政府自身建设

　　新的一年，政府工作要有新气象、新担当、新作为!我们一定牢固树立“四个意识”，坚定“四个自信”，坚决做到“两个维护”，落实全面从严治党要求，切实加强政府自身建设，深入推进政府职能转变，全面提升履职水平，为人民提供优质高效服务。

　　坚持科学民主依法全面履职。深入贯彻全面依法治国要求，严格遵守宪法，尊崇法治、敬畏法律、依法行政。自觉接受市人大及其常委会法律监督、市政协民主监督，主动接受社会和舆论监督。坚持科学、民主、依法决策，凡涉及公众利益的重大事项，都要认真听取各方意见。作为人民政府，所有工作都要体现人民意愿、维护人民利益、接受人民监督。

　　全面提高本领。大兴学习和调研之风，密切跟踪市场热点趋向、政策重点导向、科技前沿方向、资本焦点投向、人才价值取向，切实增强学习本领、政治领导本领、改革创新本领、科学发展本领、依法执政本领、群众工作本领、狠抓落实本领、驾驭风险本领，全面提升国际化、现代化、专业化水平，做到既政治过硬又本领高强。

　　勤勉干事创业。各级政府及其工作人员要以敢闯敢试、勇于改革、争创一流的拼搏精神，雷厉风行、马上就办、一抓到底的工作作风，科学理性、崇德尚法、求真务实的专业水准，干字当头、担当实干、埋头苦干，干出长春新面貌，干出人民群众好口碑。

　　保持廉洁本色。推进“两学一做”学习教育常态化制度化，认真开展“不忘初心、牢记使命”主题教育。坚决贯彻落实中央八项规定和习近平总书记在十九届中央纪委三次全会上的重要讲话精神。始终牢记习近平总书记对德惠“6·3”特大火灾事故、侵华日军“第一〇〇部队”遗址、长生问题疫苗案件作出的重要指示批示，汲取教训，举一反三，进一步提高改进作风的自觉性。持续深入、全面彻底肃清孙政才、苏荣、王珉流毒影响。巩固发展反腐败斗争压倒性胜利，坚决惩治各类腐败行为。政府工作人员要持廉守正，干干净净为人民做事，决不辜负人民公仆的称号。

　　各位代表！习近平总书记在深入推进东北振兴座谈会上的重要讲话和视察东北时的重要指示精神为我们指引了前进的方向，高质量发展的宏伟目标激励我们更加奋发有为。让我们更加紧密地团结在以习近平同志为核心的党中央周围，高举习近平新时代中国特色社会主义思想伟大旗帜，在省委、省政府和市委的坚强领导下，解放思想、团结一心，真抓实干、开拓进取，为实现长春全面振兴、全方位振兴作出新的更大贡献，以优异成绩迎接中华人民共和国成立70周年！
